# Machine Learning–Based Risk Prediction of In-Hospital Mortality in Patients With Non–ST-Elevation Acute Coronary Syndrome at Various Stages of the Diagnostic Process: Observational Study

**DOI:** 10.2196/89242

**Published:** 2026-08-19

**Authors:** Vyacheslav V Ryabov, Anastasiia K Nesova, Andrey A Vishnevskiy, Karina I Shakhgeldyan, Boris I Geltser

**Affiliations:** 1 Cardiology Research Institute, Tomsk National Research Medical Center, Russian Academy of Sciences, 111a Kievskya Street, 634012 Tomsk, Russian Federation, Tomsk, Tomsk Oblast, Russian Federation, 7 923-602-5410; 2 Department of Cardiology, Siberian State Medical University, Tomsk, Tomsk Oblast, Russian Federation; 3 Vladivostok State University, Vladivostok, Primorye, Russian Federation; 4 School of Medicine and Life Science Far Eastern Federal University, Vladivostok, Primorye, Russian Federation

**Keywords:** non–ST-elevation acute coronary syndrome, in-hospital mortality, machine learning, prognostic models, clinical decision-making

## Abstract

**Background:**

Despite advances in understanding and treating non–ST-elevation acute coronary syndrome (NSTE-ACS), patients continue to experience high rates of adverse outcomes, particularly those with non–ST-segment elevation myocardial infarction, which remains a leading cause of cardiovascular mortality. Existing risk models may not fully reflect contemporary patient populations due to substantial changes in clinical profiles. Developing new machine learning (ML)–based risk calculators may improve the prediction of in-hospital mortality (IHM) at different stages of the diagnostic process, and ultimately improve patient outcomes.

**Objective:**

This study aimed to develop predictive models for IHM in patients with NSTE-ACS using ML methods and predictor sets obtained during the diagnostic process.

**Methods:**

This retrospective observational study included 1144 patients with NSTE-ACS admitted between 2019 and 2021. IHM occurred in 94 (8.1%) of 1144 patients. Predictive models were developed using multivariable logistic regression, Random Forest, XGBoost (Extreme Gradient Boosting), and CatBoost algorithms. Model performance was evaluated using the area under the receiver operating characteristic curve (ROC-AUC), area under the precision-recall curve, calibration metrics, and decision curve analysis.

**Results:**

A key feature of the developed models was their applicability at different stages of the diagnostic process, using predictors available at each specific stage. At admission, ML models achieved ROC-AUC values up to 0.93. After incorporating laboratory and echocardiographic data, predictive performance increased to ROC-AUC values of 0.94 to 0.95. The best-performing model demonstrated an ROC-AUC of 0.951 (95% CI 0.946‐0.956), a sensitivity of 0.902 (95% CI 0.889‐0.916), and a specificity of 0.891 (95% CI 0.886‐0.896). The area under the precision-recall curve reached 0.685, and the Brier score was 0.0331, indicating good calibration. Random Forest models demonstrated greater clinical utility than the Global Registry of Acute Coronary Events score (*P*<.001). Shapley Additive Explanations analysis identified the most significant predictors of mortality risk, including Killip class of acute heart failure, age, Charlson comorbidity index, creatinine level, and hematocrit level.

**Conclusions:**

ML-based models enabled accurate prediction of IHM in patients with NSTE-ACS at different stages of the diagnostic process and may improve risk stratification and clinical decision-making in real-world practice.

## Introduction

Despite modern advances in understanding the pathophysiology of non–ST-segment elevation acute coronary syndrome (NSTE-ACS) and the continuous refinement of pharmacological and invasive treatment strategies, registry studies continue to report a high incidence of adverse outcomes in the aforementioned patient population, with no discernible trend toward reduction in recent years [1,2]. The well-known heterogeneity of the population of patients with NSTE-ACS presents a major challenge in making optimal clinical decisions [3,4]. Furthermore, non–ST-segment elevation myocardial infarction (NSTEMI), which is a primary nosological entity within NSTE-ACS, remains a leading cause of morbidity and mortality among cardiovascular diseases (CVDs) worldwide [5]. The insufficient improvement in both short- and long-term outcomes for patients with NSTEMI compared to those with ST-segment elevation myocardial infarction (STEMI) is likely attributable to the more complex clinical phenotype of the former group [6].

Given the marked heterogeneity of the clinical profiles of patients with NSTE-ACS, the refinement of risk stratification for predicting adverse cardiovascular events, including in-hospital mortality (IHM), is an integral component of optimizing diagnostic and treatment strategies to improve prognosis [7,8]. To date, the Global Registry of Acute Coronary Events (GRACE) risk calculator remains the only guideline-recommended scoring system for assessing the risk of adverse outcomes in patients with NSTE-ACS [9-12]. However, GRACE was developed and validated in the early 2000s and is therefore based on clinical and demographic data that may not fully reflect the contemporary patient population [13,14]. Over the past decade, the clinical profile of patients with NSTE-ACS has evolved substantially due to population aging and the increasing prevalence of comorbidities. This likely affects the applicability of existing risk calculators both in specific subgroups, such as female and older patients [15], and in the contemporary NSTE-ACS population overall [16].

Moreover, traditional statistical models used in most risk scores, including logistic regression, may have limitations when handling a large number of predictors and complex nonlinear relationships between variables and outcomes. In this context, machine learning (ML) approaches have attracted increasing attention because of their ability to model complex interactions and potentially improve predictive performance [17].

Recent studies have demonstrated the potential of ML methods for predicting IHM in patients with NSTEMI or NSTE-ACS. Algorithms such as Random Forest (RF), XGBoost (Extreme Gradient Boosting), LightGBM, support vector machines, and deep learning have shown improved discrimination compared with traditional risk scores. However, existing studies remain heterogeneous with respect to patient populations, data sources, and clinical settings, including intensive care unit–based datasets [18], NSTEMI-only cohorts [19], or NSTE-ACS populations restricted to patients undergoing percutaneous coronary intervention [20]. Importantly, relatively few studies have evaluated ML-based risk prediction models designed for use at different stages of the diagnostic process in a contemporary, real-world NSTE-ACS population.

This study was designed to address these limitations. We developed ML models to predict IHM in a contemporary real-world cohort of patients with NSTE-ACS. Unlike many previous studies, the proposed models were designed for application at different stages of the diagnostic process, including the stage before laboratory data become available. The performance of several ML algorithms was compared with traditional approaches, including logistic regression and the GRACE-based strategy, with the aim of developing clinically applicable risk prediction tools for NSTE-ACS management.

## Methods

### Overview

**Figure 1.**
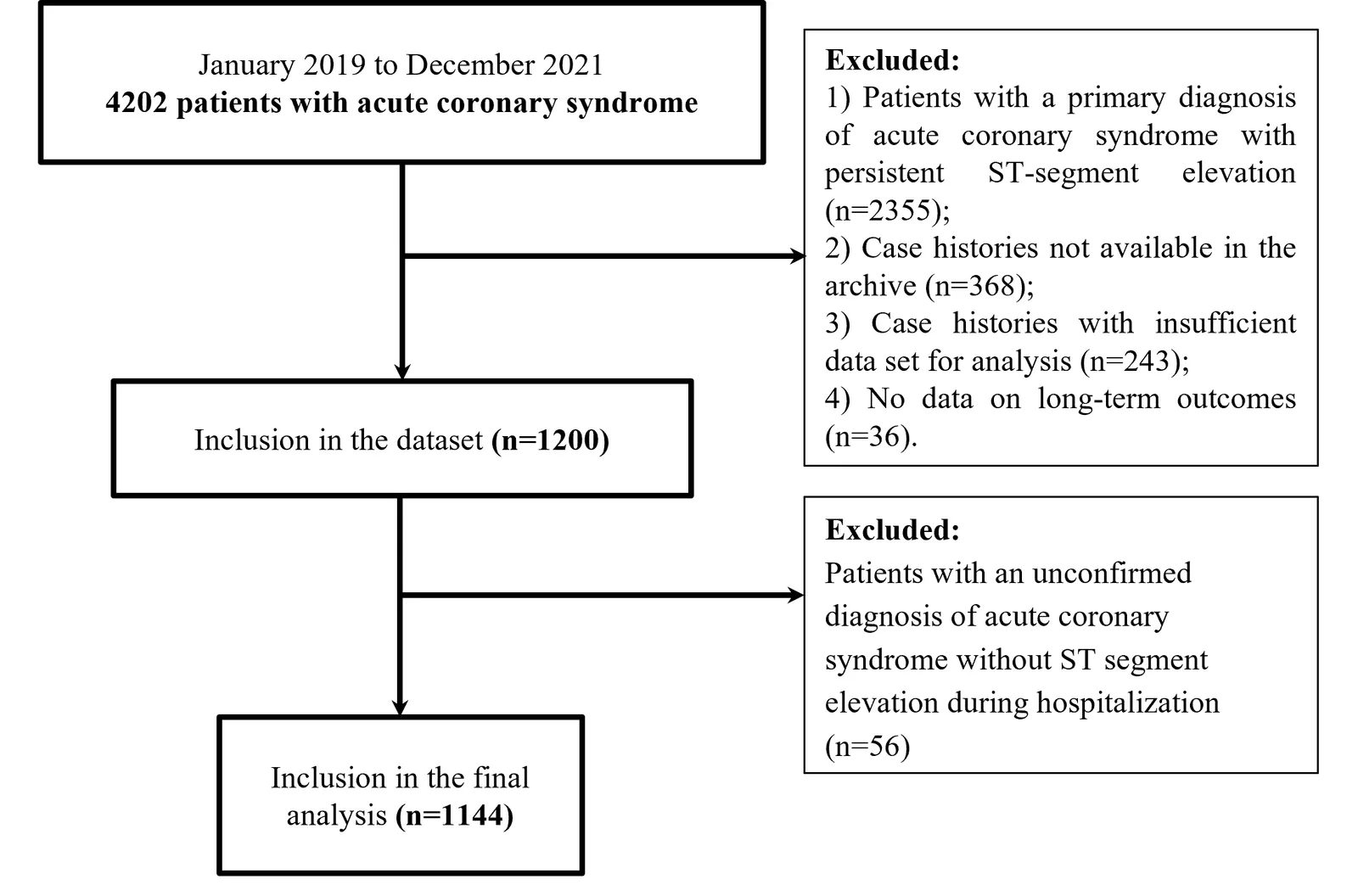
Flowchart of patients enrolled.

This study used data acquired at the Research Institute of Cardiology, a branch of the Federal State Budgetary Scientific Institution “Tomsk National Research Medical Center of the Russian Academy of Sciences.” The anonymized dataset included information on 1144 patients with NSTE-ACS (men: n=657, 57.4%; and women: n=487, 42.6%), aged 30 to 98 (median 67, IQR 59.7-75) years, who were admitted to the facility between 2019 and 2021. All patients included in the study signed written informed consent for the processing of personal data. The final cohort consisted of 512 patients with a clinical diagnosis of unstable angina and 632 patients with primary or recurrent NSTEMI. The patient enrollment flowchart is presented in Figure 1.

### Eligibility Criteria

#### Inclusion Criteria

The inclusion criteria were as follows:

- Diagnosis at admission: NSTE-ACS, defined as the presence of acute clinical signs or symptoms of myocardial ischemia, with electrocardiography (ECG) showing no persistent ST-segment elevation in at least two adjacent leads and no new-onset left bundle branch block
- Patients of any gender aged ≥18 years
- Consent to the processing of personal data signed by the patient or their legal representative upon admission

#### Exclusion Criteria

The exclusion criteria were as follows:

- Patients with an initial diagnosis of acute coronary syndrome with persistent ST-segment elevation
- Lack of clinical and diagnostic data necessary to make a meaningful assessment of the condition and to study disease outcomes
- No consent for the processing of personal data, signed by the patient or their legal representative

### Data Collection

For each patient, data on 149 parameters were retrospectively collected, encompassing anamnestic, anthropometric, clinical, and laboratory characteristics (Table S1 in Multimedia Appendix 1). Data were stored in a personalized electronic database using Microsoft Excel 2010 (Certificate of State Registration 2023622190 dated July 3, 2023) [21]. Demographic and anthropometric data included age, sex, and BMI. The anamnestic information analyzed as predictors of adverse events included the functional class of chronic heart failure (CHF), prior decompensation of CHF, and a history of conditions such as anemia or bleeding, uncontrolled arterial hypertension, essential arterial hypertension, hypotensive episodes (blood pressure <90/60 mm Hg), pulmonary embolism, peritonitis, coronary artery disease (CAD), atrial fibrillation, acute cerebrovascular events, and type 2 diabetes mellitus. Additional variables included were the presence and stage of chronic kidney disease (CKD), the presence of malignant neoplasm, peripheral arterial disease, a family history of CVD, duration of CAD, the number of prior myocardial infarctions, the number of previously implanted intracoronary stents, and the Charlson comorbidity index (CCI). Furthermore, the dataset captured prior procedures, such as history of invasive coronary angiography (ICA), percutaneous coronary intervention, and coronary artery bypass grafting. Indicators characterizing the patients’ condition at admission were also assessed, such as the presence of community-acquired pneumonia (CAP), class of acute heart failure (AHF) by T. Killip (Killip class), anginal pain at admission, positive response to short-acting nitrates, pain radiation, dyspnea on admission, palpitations, symptoms of weakness, dizziness, systolic blood pressure (SBP), diastolic blood pressure (DBP), heart rate (HR), pulse, pulmonary rales and dry rales, signs of ongoing myocardial ischemia on admission, CRUSADE (Can Rapid risk Stratification of Unstable Angina Patients Suppress Adverse Outcomes with Early Implementation) score, cardiac arrhythmias, and oxygen saturation level.

Within the first hours of hospitalization, a standard set of laboratory tests was performed, assessing the following parameters: white blood cell count, hemoglobin level, red blood cell count, hematocrit, platelet count, glucose, creatinine, total bilirubin, alanine aminotransferase, aspartate aminotransferase, creatine phosphokinase, creatine phosphokinase-MB, total cholesterol, triglycerides, fibrinogen levels, as well as the international normalized ratio.

Data collection spanned a 3-year period (2019-2021). During this time, 2 different immunoassay analyzers (with different reference intervals) were used in the hospital to determine troponin I levels: “Access2” (upper limit of normal [99th percentile] of 0.04 ng/mL) and “AQT90 FLEX” (upper limit of normal [99th percentile] of 0.023 ng/mL). Consequently, quantitative data for this marker were not included in the subsequent analysis; instead, the fact of troponin I elevation above the 99th percentile was used.

Within the first hours of hospitalization, the following instrumental studies were performed: ECG was used to assess the QRS duration, QT and corrected QT (QTc) intervals, and the presence of pathological Q waves. Echocardiography (EchoCG) was performed to measure the longitudinal dimensions of the left atrium and right ventricle, thickness of the interventricular septum and posterior wall of the left ventricle, myocardial mass and mass index, end-diastolic and end-systolic dimensions and volumes, stroke volume (SV), the peak early (E) to late (A) ventricular filling velocity ratio (E/A), and left ventricular ejection fraction (LVEF) using both M-mode and B-mode.

For each patient, the glomerular filtration rate was calculated during the hospital stay, along with the GRACE risk score. Among the indicators describing the dynamics of the patient’s condition, the development of hospital-acquired pneumonia (HAP) was additionally considered. All clinical characteristics and factors assessed during hospitalization occurred prior to the IHM and were considered predictors rather than perimortem events.

Additionally, data obtained during ICA were analyzed. ICA was performed during the index hospitalization in 900 (78.7%) of 1144 patients. The presence of multivessel atherosclerotic CAD (defined as ≥2 major epicardial arteries with a diameter of >2.5 mm affected) and angiographic evidence of slow coronary flow were used as potential prognostic factors.

All binary indicators, describing the presence or absence of a characteristic, were coded as 1 (present) or 0 (absent). Variables with multiple levels, such as Killip class of AHF, New York Heart Association functional class, and CKD stage, were transformed into several variables. The first variable was modeled as a continuous measure (values 0‐4), the second as a dichotomous variable indicating the presence or absence of the condition (AHF, CKD, or CHF), while several additional variables were constructed to contrast mild versus severe disease stages (CKD stages 3‐5 coded as “1,” otherwise “0”; Killip classes III and IV of AHF coded as “1,” otherwise “0”).

The primary end point of the study was the occurrence of IHM or death within 30 days of the acute coronary event. Hereafter, this composite end point is referred to as IHM and is coded as “1.” A code of “0” was assigned to survivors, defined as patients who were alive at discharge and at 30 days after the onset of NSTE-ACS.

**Figure 2.**
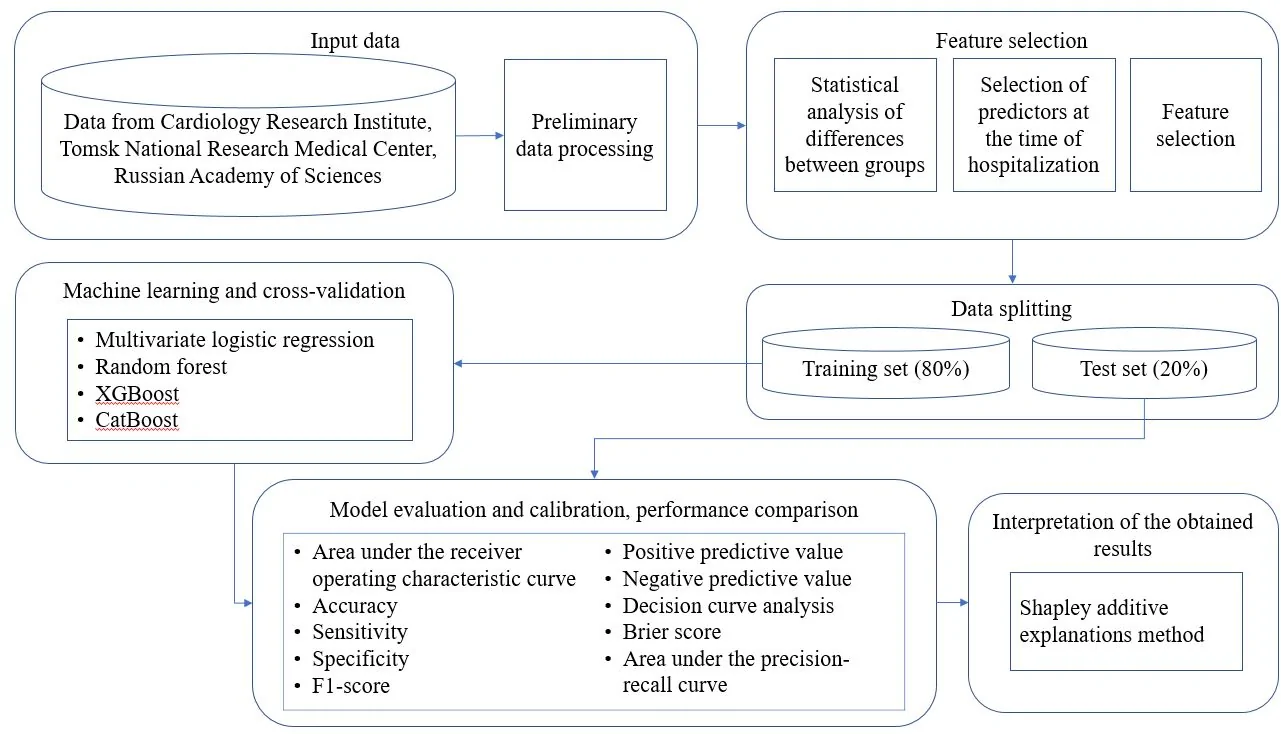
Study design flowchart.

It should be noted that the data obtained on the frequency of IHM do not fully reflect the institution’s overall mortality rates due to the flow-based processing of information and the exclusion of a number of patients from this study for the reasons mentioned earlier (Figure 1). The study included 6 main stages (Figure 2).

At stage 1, data were cleaned to remove manual entry errors, while categorical features were encoded. Missing data were not imputed. If the missingness of a variable exceeded 10%, such variables were excluded from further analysis (Table S2 in Multimedia Appendix 1). The missing data were mainly attributable to the heterogeneity of patients’ medical history at the time of hospital admission, as well as to differences in the clinical indication for assessing certain laboratory parameters depending on the patient’s clinical status (eg, presence or absence of anemia or diabetes mellitus) and during follow-up measurements (eg, hemoglobin and creatinine levels). Variables that demonstrated statistically significant differences but were not included in the final prognostic models contained missing values ranging from 0% to 10%, which were not imputed. Continuous variables were presented as median and IQR (Q1; Q3), while categorical variables were presented as counts and percentages.

At the second stage, statistical analysis of the data was performed, comprising an intergroup comparison of 149 continuous and categorical features.

At the third stage, using the data normalized in the previous stage by z-score standardization (mean 0, SD 1), univariate logistic regression (ULR) was applied to calculate weight coefficients and area under the receiver operating characteristic curve (ROC-AUC) with 95% CIs for a preliminary assessment of potential predictors. The ULR model was trained on 80% of the data randomly selected from the dataset. The ROC-AUC estimate was obtained by testing the predictive ability of the ULR model on the final test set, which contained the remaining 20% of the data. The procedure of splitting the data into 80% and 20% subsets was repeated 100 times with the computation of the ULR model, weight coefficients, and ROC-AUC; the results were averaged, and the 95% CI was estimated.

During the fourth phase of the study, predictive models for IHM were developed using the following ML algorithms: multivariate logistic regression (MLR), RF, Stochastic Gradient Boosting (XGBoost), and CatBoost (CB).

The models were designed to reflect several distinct stages of the diagnostic workflow for patients with NSTE-ACS. The first scenario used data available at initial presentation, including demographic, anthropometric, and anamnestic data, as well as findings from the physical examination. CAP was modeled separately, as its diagnosis requires additional confirmatory testing.

The second diagnostic scenario incorporated laboratory results. The third scenario added findings from instrumental investigations, specifically EchoCG. The final, fourth scenario accounted for the potential development of HAP during hospitalization.

For baseline comparison, a ULR model was also calculated based on a single predictor—the GRACE risk score.

At the fifth stage, the Shapley Additive Explanations (SHAP) method [22] was used to interpret the output of the best-performing model. Furthermore, the clinical utility of the models was evaluated using decision curve analysis (DCA) [23].

The normality of continuous variables was assessed using the Kolmogorov-Smirnov test. As none of the variables followed a normal distribution, the Mann-Whitney *U* test was used for intergroup comparisons. Differences in the frequency of categorical variables were analyzed using the chi-square test. A *P* value of less than .05 was considered statistically significant. For dichotomous variables, the Fisher exact test was additionally used to calculate odds ratios (ORs) with 95% CI.

Potential predictors for the prognostic model were identified through forward sequential feature selection using both ULR and MLR algorithms. Model performance was evaluated using stratified 10-fold cross-validation. The optimization metric for hyperparameter tuning was the maximization of the ROC-AUC.

### ML Algorithms

For training, cross-validation, and final testing of all multivariable prognostic models, the dataset was randomly split as follows: 80% of the data were used for training and cross-validation, performed using stratified 10-fold cross-validation, and 20% were reserved for final testing.

Model parameter and hyperparameter tuning were performed to maximize the ROC-AUC during cross-validation. The final model quality was assessed on the test set using the ROC-AUC and additional metrics, including accuracy, sensitivity, specificity, and the *F*_1_-score. To calculate these latter metrics, an optimal probability threshold was determined by identifying the point that maximized the average of sensitivity and specificity across the cross-validation folds. Given the class imbalance (with an IHM prevalence of 8.1%), the Brier score was calculated for all models [24].

The selection of predictors for the multivariate models was guided by the ROC-AUC values from the ULR models. A feature was included in the pool of potential predictors for IHM if it increased the cross-validated ROC-AUC of the multivariate model. Predictors selected by MLR were subsequently used to train the RF, XGBoost, and CB models. For these ensemble methods, the possibility of including other potential predictors was also evaluated.

Hyperparameter tuning was performed at the same stage. To estimate the 95% CIs of the model performance metrics, the random data-splitting procedure (80% training and cross-validation, 20% testing) was repeated 100 times; the results were averaged, and 95% CIs were calculated. This approach minimized the risk of an “optimistic” test set selection. To compare the predictive performance of the models, a bootstrap procedure with 1000 resamples, identical for the compared models, was applied, and *P* values were estimated.

For the best-performing models, area under the precision-recall curve (PR-AUC) and model calibration were assessed. PR-AUC was calculated using average precision, defined as the weighted mean of precision at each threshold, with the increase in recall serving as the weight. Calibration analysis was conducted for 3 models corresponding to different stages of the diagnostic process. Models were trained on 80% of the data, while calibration curves were evaluated on the remaining 20% test dataset. For the best-performing model, the relationship between positive predictive value (PPV) and negative predictive value (NPV) across classification thresholds was also analyzed.

Predictor importance for the best-performing model was assessed using SHAP values. Finally, all models, including the GRACE score baseline model, were compared for their clinical utility using DCA. Data analysis was performed using the Python programming language and the following libraries: NumPy, SciPy, Pandas, CB, XGBoost, Sklearn, DCA, and SHAP.

### Ethical Considerations

This study was conducted in accordance with the principles of the Declaration of Helsinki and was approved by the Human Research Ethics Committee of the Research Institute of Cardiology, Tomsk National Research Medical Center (protocol No. 235, November 23, 2022). The study was based on retrospectively collected clinical data obtained during routine medical care. Upon hospital admission, patients provided written consent for the processing of their personal data in accordance with institutional policy and applicable legislation. Before analysis, all data were deidentified to protect participants' privacy and confidentiality. No additional interventions or direct contact with participants were performed as part of this study. As this was a retrospective observational study, prospective registration was not required under the International Committee of Medical Journal Editors recommendations.

## Results

### Overview

Of the 1144 patients with NSTE-ACS included in the study, IHM was recorded in 94 (8.1%) of 1144 patients. The group with fatal outcomes demonstrated a significantly higher prevalence of specific clinical and laboratory characteristics (Table S1 in Multimedia Appendix 1).

Patients in the IHM group were significantly older, with a median age of 80.5 (IQR 72‐85) years compared to 66 (IQR 60‐75) years in the survivor group (*P*<.001), while the absence of a family history of CVD was significantly more common (78/94, 83% vs 611/1050, 58.2%; *P*<.001). BMI was significantly higher in survivors than in nonsurvivors (median 28.6, IQR 25.5-32.4 vs 26, IQR 22.9-29.2; *P*<.001).

Significant differences were observed in clinical presentation at admission. Weakness was reported by 37 (39%) of 94 patients in the IHM group versus 172 (16.4%) of 1050 patients in the survivor group (OR 3.3, 95% CI 2.1‐5.2; *P*<.001). Loss of consciousness was recorded in 14 (15%) of 94 patients in the IHM group compared to 20 (2%) of 1050 patients among survivors (OR 9.0, 95% CI 4.4‐18.5; *P*<.001).

SBP was significantly lower in the IHM group, with a median of 110.5 mm Hg (IQR 99.3‐135.8) versus 139.5 mm Hg (IQR 123‐155) in survivors (*P*<.001). Similar differences were recorded for HR (median 90, IQR 75‐109 bpm vs 75, IQR 65‐88 bpm; *P*<.001). The presence of auscultative phenomena was strongly associated with mortality. Both dry (11/94, 12% vs 42/1050, 4%; *P*=.002) and pulmonary (36/94, 38% vs 110/1050, 10.5%; *P*<.001) rales were significantly more frequent in the IHM group.

Overall, the CCI was significantly higher in the mortality group (median 8, IQR 6-10 vs 5, IQR 3-7; *P*<.001). Conversely, an inverse association was observed for certain traditional risk factors. The proportion of active smokers was lower in the IHM group (24/94, 26%) compared to survivors (446/1050, 42.5%; *P*<.001), as was the prevalence of a family history of CVD (16/94, 17% vs 439/1050, 41.8%; *P*<.001). Comorbidities and complications during hospitalization also played a crucial role. CAP was present in 17 (18%) of 94 patients in the IHM group compared to 25 (2%) of 1050 patients among survivors (OR 9.1, 95% CI 4.7‐17.5; *P*<.001). Cardiogenic shock was observed more frequently in the nonsurvivor group than in the survivor group (41/94, 44% vs 13/1050, 1%; OR 61.7, 95% CI 31.2‐122.1; *P*<.001). Similarly, AHF Killip classes II and III were more common among nonsurvivors (63/94, 67%) than among survivors (112/1050, 10.7%; OR 29.8, 95% CI 18.2‐49.0; *P*<.001).

Laboratory findings also revealed significant disparities between the groups. Hemoglobin levels were markedly lower in the nonsurvivor group, with a median of 113 (IQR 99‐132) g/L compared to 136 (IQR 123‐148) g/L in survivors (*P*<.001). Hematocrit levels were similarly reduced, measuring 34% (IQR 30‐39) versus 39.9% (IQR 36‐43) in the survivor group (*P*<.001). Conversely, creatinine levels were significantly elevated in patients with fatal outcomes, registering a median of 129 (IQR 101‐189) μmol/L against 94 (IQR 81‐115) μmol/L in survivors (*P*<.001).

A number of instrumental findings from ECG and EchoCG also demonstrated statistically significant differences between the groups. The most substantial disparities were observed for the following parameters. LVEF was significantly lower in nonsurvivors (median 44%, IQR 35‐59) than in survivors (median 60%, IQR 52‐64*; P*<.001). Furthermore, left ventricular systolic dysfunction (LVSD; LVEF <49%) was observed in 44 (47%) of 94 patients in the IHM group compared to 171 (16.3%) of 1050 patients in the survivor group (*P*<.001). SV was substantially reduced in nonsurvivors: 45.5 (IQR 36.6‐55) mL versus 60 (IQR 51‐69) mL in the survivor group (*P*<.001). Multivessel CAD was associated with a significantly higher incidence of fatal outcomes (42/94, 45% vs 303/1050, 28.9%; *P*<.001). The median QTc interval in the fatal outcome group was 433 (IQR 413‐461) ms compared to 419 (IQR 402‐439) ms in survivors (*P*=.001). The QRS duration was also longer: 108 (IQR 94‐130) ms versus 98 (IQR 88‐110) ms (*P*<.001).

The results of the ULR analysis, performed on features with statistically significant intergroup differences, demonstrate significant associations between various patient characteristics and IHM and allow for the ranking of these features by their level of importance (Table S3 in Multimedia Appendix 1). On the basis of the ULR analysis, the presence of AHF demonstrated the highest prognostic significance for IHM (ROC-AUC=0.805; β=4.6). It was closely followed by the glomerular filtration rate (ROC-AUC=0.799; β=−4.95), age (ROC-AUC=0.793; β=4.52), LVSD (ROC-AUC=0.786; β=3.1), SV (ROC-AUC=0.774; β=−3.6), and oxygen saturation level (ROC-AUC=0.774; β=−4.03). Several other variables also showed high prognostic potential (ROC-AUC>0.7), including the CCI, SBP, DBP, LVEF, troponin-I, CRP levels, hemoglobin and hematocrit levels, the presence of multivessel CAD, and signs of ongoing myocardial ischemia.

### Model Development

At the third stage, prognostic models for IHM were developed corresponding to the following diagnostic scenarios:

1. At hospital admission, prior to the availability of laboratory results and the ability to calculate the GRACE risk score, which requires creatinine and troponin I levels (models 1 and 2)
2. After obtaining laboratory results
3. After obtaining EchoCG results
4. During hospitalization

The validation and testing results of the MLR prognostic models across these different stages of the diagnostic and treatment process are presented in Table 1.

**Table 1.** Performance evaluation of multivariate logistic regression prognostic models on cross-validation and final testing at different stages of the diagnostic process.

Treatment stage and model	Predictors	ROC-AUC^a^ during cross-validation	ROC-AUC during final testing
Admission			
1	Age, Killip class of acute heart failure, systolic blood pressure, heart rate, BMI, symptoms of weakness, Charlson comorbidity index, family history of cardiovascular diseases, dry rales, loss of consciousness	0.920 (0.918, 0.921)	0.916 (0.910, 0.922)
2	Age, Killip class of acute heart failure, systolic blood pressure, heart rate, BMI, symptoms of weakness, Charlson comorbidity index, family history of cardiovascular diseases, dry rales, loss of consciousness, community-acquired pneumonia	0.921 (0.920, 0.923)	0.917 (0.911, 0.923)
After laboratory tests			
3	GRACE^b^	0.911 (0.910, 0.913)	0.907 (0.901, 0.913)
4	Age, systolic blood pressure, heart rate, Killip class of acute heart failure, creatinine	0.888 (0.886, 0.890)	0.889 (0.881, 0.897)
5	Age, systolic blood pressure, heart rate, Killip class of acute heart failure, creatinine, hematocrit, BMI, community-acquired pneumonia, symptoms of weakness, Charlson comorbidity index, family history of cardiovascular diseases, dry rales, loss of consciousness	0.923 (0.922, 0.925)	0.926 (0.921, 0.931)
6	GRACE, hematocrit, BMI, community-acquired pneumonia, symptoms of weakness, Charlson comorbidity index, family history of cardiovascular diseases, dry rales, loss of consciousness	0.928 (0.927, 0.930)	0.928 (0.923, 0.934)
After receiving the echocardiography data			
7	GRACE, hematocrit, BMI, community-acquired pneumonia, symptoms of weakness, Charlson comorbidity index, family history of cardiovascular diseases, dry rales, loss of consciousness, left ventricular systolic dysfunction	0.928 (0.927, 0.930)	0.935 (0.929, 0.940)
After receiving the echocardiography data			
8	GRACE, hematocrit, BMI, community-acquired pneumonia, symptoms of weakness, Charlson comorbidity index, family history of cardiovascular diseases, dry rales, loss of consciousness, left ventricular systolic dysfunction, hospital-acquired pneumonia	0.933 (0.932, 0.935)	0.940 (0.934, 0.945)
9	GRACE, hematocrit, BMI, community-acquired pneumonia, symptoms of weakness, Charlson comorbidity index, family history of cardiovascular diseases, dry rales, loss of consciousness, left ventricular systolic dysfunction, hospital-acquired pneumonia, cardiogenic shock	0.940 (0.939, 0.942)	0.947 (0.942, 0.953)

a ROC-AUC: area under the receiver operating characteristic curve.

b GRACE: Global Registry of Acute Coronary Events.

Models 1 and 2 differ by the inclusion of the CAP feature in the latter. The performance evaluation of models 1 and 2 during cross-validation and final testing shows their minor differences, with the latter demonstrating superior metrics.

Following the assessment of laboratory results, models 3 to 6 can be computed. The baseline model based on the GRACE risk score showed higher prognostic accuracy than a model using its component predictors in a continuous form (ROC-AUC=0.907 vs 0.889).

The inclusion of additional predictors—such as anthropometric data (BMI), anamnestic data (CCI and family history of CVD), assessment of the patient’s condition at admission (weakness, dry rales, and loss of consciousness), clinical data, and hematocrit level—improved prognostic accuracy compared to the GRACE score alone, achieving an ROC-AUC of 0.928. Using the LVSD predictor further increased performance to an ROC-AUC of 0.935. Finally, accounting for the development of HAP and cardiogenic shock during treatment yielded the highest accuracy, with an ROC-AUC of 0.947.

Using various ML methods, prognostic models for IHM in patients with NSTE-ACS were developed (Table 2).

**Figure 3.**
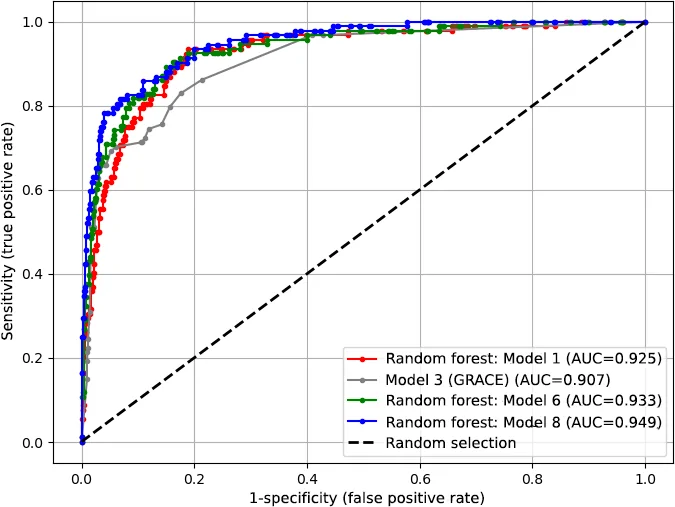
The best in-hospital mortality models in patients with non–ST-elevation acute coronary syndrome for different stages of the diagnostic process. AUC: area under the curve; GRACE: Global Registry of Acute Coronary Events.

**Figure 4.**
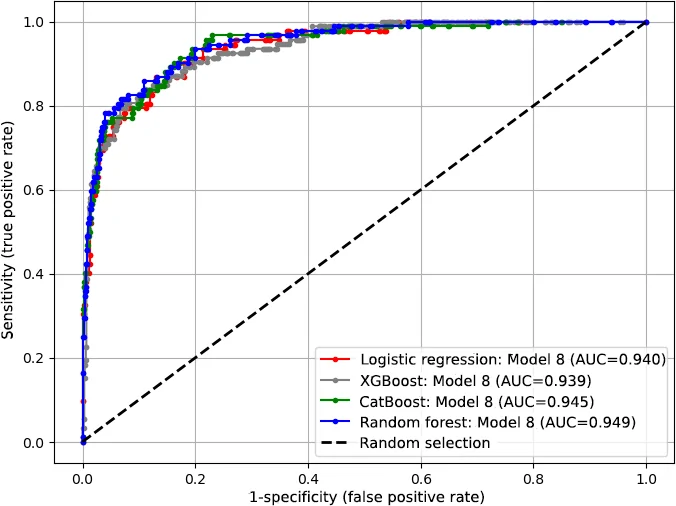
Comparison of receiver operating characteristic curve graphs for models developed using different machine learning methods. AUC: area under the curve; XGBoost, Extreme Gradient Boosting.

The analysis of the developed predictive models revealed that all of them exhibited high forecasting accuracy, which ranged from an ROC-AUC of 0.889 (for the first model that used isolated predictors from the GRACE scale) to 0.951 (for the model that included, in addition to the GRACE predictors, features assessed at each stage of the diagnostic and therapeutic process). Models developed using various ML methods demonstrated a consistent increase in predictive value with the inclusion of predictors selected at subsequent stages of the diagnostic process (Figure 3). The best models at each stage were created using RF (Figure 4).

**Table 2.** Performance metrics of in-hospital mortality prognostic models in patients with non–ST-segment elevation acute coronary syndrome developed using machine learning methods.

Model	Threshold	AUC^a^, estimate (95% CI)	Accuracy, estimate (95% CI)	Sensitivity, estimate (95% CI)	Specificity, estimate (95% CI)	*F*_1_-score, estimate (95% CI)
MLR^b^ (L2 regularization)						
1	0.075	0.916 (0.910-0.922)	0.847 (0.842-0.851)	0.836 (0.821-0.852)	0.847 (0.842-0.852)	0.476 (0.468-0.485)
2	0.075	0.917 (0.911-0.923)	0.849 (0.845-0.853)	0.833 (0.817-0.849)	0.850 (0.846-0.855)	0.479 (0.471-0.487)
3	0.083	0.907 (0.901-0.913)	0.817 (0.812-0.821)	0.799 (0.784-0.815)	0.818 (0.813-0.824)	0.421 (0.414-0.429)
4	0.058	0.889 (0.881-0.897)	0.813 (0.808-0.819)	0.814 (0.798-0.831)	0.813 (0.807-0.820)	0.422 (0.414-0.430)
5	0.074	0.926 (0.921-0.931)	0.850 (0.846-0.854)	0.847 (0.833-0.861)	0.851 (0.846-0.856)	0.487 (0.480-0.494)
6	0.1	0.928 (0.923-0.934)	0.863 (0.858-0.867)	0.855 (0.841-0.868)	0.863 (0.859-0.868)	0.511 (0.501-0.521)
7	0.1	0.935 (0.929-0.940)	0.868 (0.864-0.873)	0.872 (0.855-0.888)	0.868 (0.863-0.873)	0.514 (0.502-0.525)
8	0.095	0.940 (0.934-0.945)	0.874 (0.870-0.879)	0.884 (0.870-0.899)	0.874 (0.869-0.878)	0.529 (0.519-0.540)
9^c^	0.08	0.947 (0.942-0.953)	0.881 (0.877-0.886)	0.897 (0.884-0.911)	0.880 (0.875-0.885)	0.548 (0.537-0.558)
RandomForest: number of trees=100, maximum depth of each tree=10, bootstrap enabled, minimum 2 samples per leaf, and minimum 2 samples required to split a node.						
1	0.08	0.925 (0.920-0.930)	0.841 (0.836-0.845)	0.842 (0.827-0.856)	0.840 (0.835-0.846)	0.469 (0.460-0.478)
2	0.087	0.930 (0.925-0.935)	0.851 (0.846-0.856)	0.843 (0.828-0.859)	0.852 (0.847-0.857)	0.486 (0.476-0.496)
6^c^	0.098	0.933 (0.928-0.938)	0.867 (0.863-0.871)	0.861 (0.845-0.876)	0.868 (0.864-0.873)	0.522 (0.512-0.531)
7^c^	0.101	0.942 (0.937-0.947)	0.868 (0.863-0.873)	0.871 (0.856-0.887)	0.868 (0.863-0.873)	0.513 (0.503-0.523)
8^c^	0.11	0.949 (0.945-0.954)	0.885 (0.881-0.889)	0.887 (0.872-0.902)	0.885 (0.880-0.889)	0.551 (0.541-0.562)
9^c^	0.1	0.951 (0.946-0.956)	0.892 (0.888-0.896)	0.902 (0.889-0.916)	0.891 (0.886-0.896)	0.572 (0.561-0.583)
XGBoost learning_rate=0.1, max_depth=3, n_estimators=100, colsample_bytree=0.8, gamma=0.2, subsample=0.8						
1	0.06	0.919 (0.913-0.925)	0.843 (0.839-0.847)	0.840 (0.823-0.857)	0.843 (0.839-0.848)	0.472 (0.463-0.481)
2	0.06	0.920 (0.914-0.926)	0.844 (0.839-0.849)	0.840 (0.824-0.856)	0.844 (0.839-0.850)	0.474 (0.464-0.484)
6	0.065	0.922 (0.916-0.927)	0.841 (0.836-0.846)	0.840 (0.823-0.857)	0.841 (0.836-0.847)	0.469 (0.460-0.478)
7	0.065	0.927 (0.922-0.933)	0.842 (0.837-0.847)	0.848 (0.830-0.865)	0.842 (0.836-0.848)	0.461 (0.452-0.471)
8^c^	0.065	0.939 (0.934-0.944)	0.876 (0.872-0.881)	0.879 (0.865-0.894)	0.876 (0.871-0.881)	0.532 (0.522-0.543)
9^c^	0.06	0.941 (0.936-0.946)	0.881 (0.876-0.885)	0.878 (0.863-0.892)	0.881 (0.876-0.886)	0.541 (0.531-0.551)
CatBoost learning_rate=0.1, depth=5‐9, iterations=100, l2_leaf_reg=9						
1	0.058	0.923 (0.917-0.928)	0.845 (0.840-0.849)	0.843 (0.826-0.859)	0.845 (0.840-0.850)	0.475 (0.466-0.484)
2	0.058	0.926 (0.920-0.931)	0.852 (0.847-0.857)	0.848 (0.833-0.863)	0.852 (0.847-0.858)	0.490 (0.480-0.499)
6^c^	0.075	0.932 (0.927-0.937)	0.859 (0.854-0.863)	0.845 (0.831-0.859)	0.860 (0.855-0.865)	0.502 (0.493-0.512)
7^c^	0.105	0.934 (0.929-0.940)	0.855 (0.850-0.860)	0.856 (0.838-0.873)	0.855 (0.849-0.861)	0.486 (0.475-0.496)
8	0.103	0.945 (0.939-0.950)	0.879 (0.874-0.884)	0.876 (0.860-0.892)	0.879 (0.874-0.884)	0.536 (0.525-0.548)
9	0.095	0.947 (0.942-0.952)	0.887 (0.883-0.892)	0.878 (0.862-0.894)	0.888 (0.883-0.893)	0.555 (0.544-0.565)

a AUC: area under the curve.

b MLR: multivariate logistic regression.

c The model uses isolated predictors instead of the GRACE scale: age, heart rate, systolic blood pressure, Killip class of acute heart failure, and creatinine.

The developed models demonstrated high discriminative performance (Table 2). However, due to the pronounced class imbalance in the dataset—reflected in the expectedly low *F*_1_-score values (0.421‐0.555)—additional model calibration was performed.

PR-AUC analysis accounting for class imbalance (IHM prevalence of 8.1%) demonstrated good predictive performance of the developed models. Model 1, predicting IHM at hospital admission, achieved a PR-AUC of 0.620 (95% CI 0.613‐0.627). Model 6, developed for mortality prediction when laboratory data were available, achieved a PR-AUC of 0.641 (95% CI 0.634‐0.647). Model 7, incorporating echocardiographic parameters, showed a PR-AUC of 0.647 (95% CI 0.640‐0.653). Model 9, predicting IHM in the postoperative period, demonstrated the highest PR-AUC of 0.685 (95% CI 0.679‐0.690).

**Figure 5.**
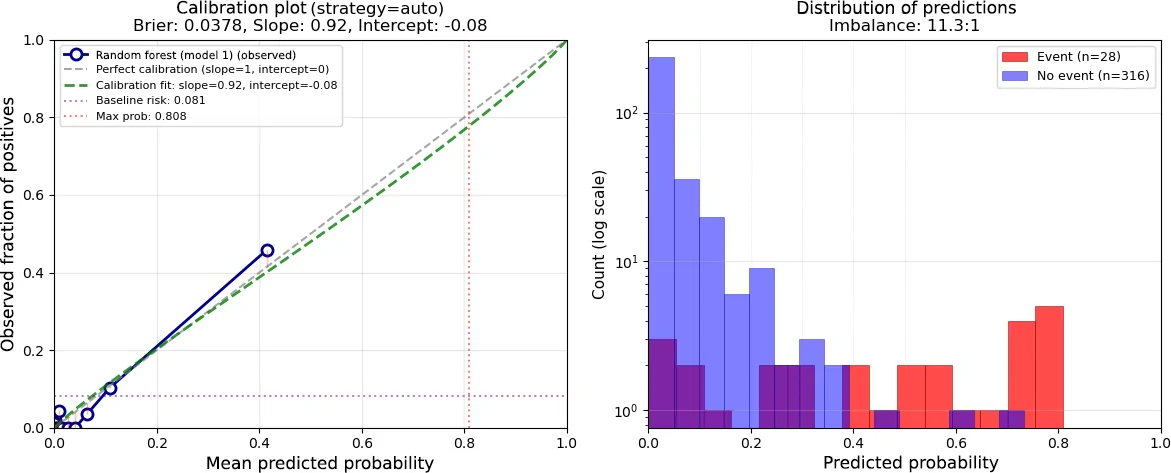
Calibration curve for model 1 based on predictions from the test dataset.

**Figure 6.**
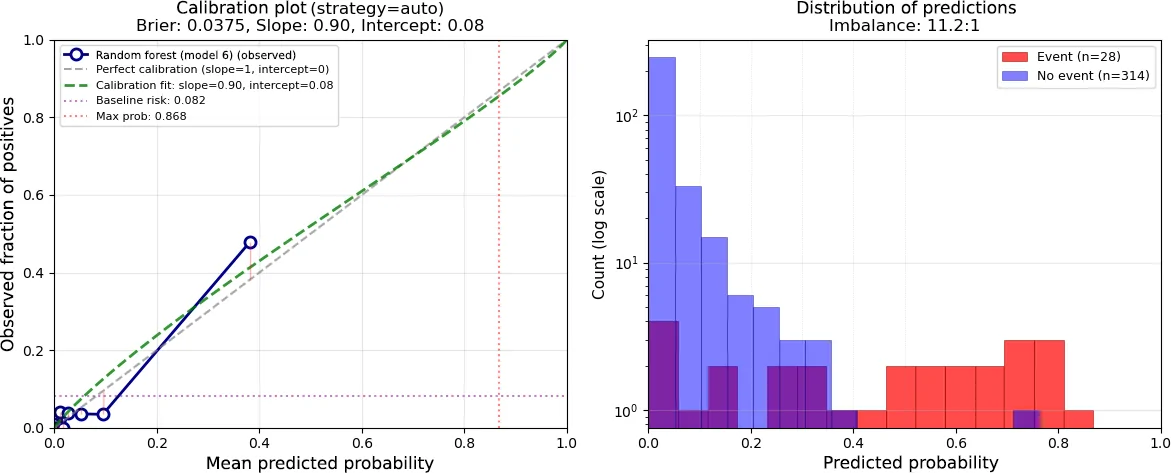
Calibration curve for model 6 based on predictions from the test dataset.

**Figure 7.**
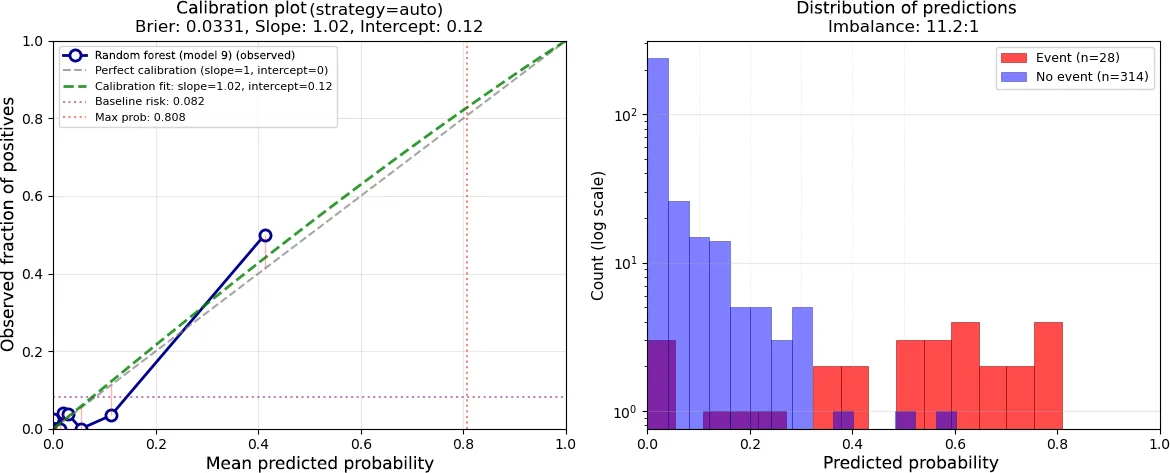
Calibration curve for model 9 based on predictions from the test dataset.

Calibration curves were constructed for models 1, 6, and 9, which were trained on 70% of the data and evaluated on the remaining 30% test dataset (Figures 5-7). Analysis of the calibration curves demonstrated a high level of agreement between predicted and observed outcomes. The predicted outcome points were located close to the line of ideal calibration. The Brier scores were 0.0378, 0.0375, and 0.033, indicating excellent model calibration, given the prevalence of IHM of approximately 8.1%. Figures 5-7 also present the distribution of predicted probabilities for the groups of deceased and surviving patients.

**Figure 8.**
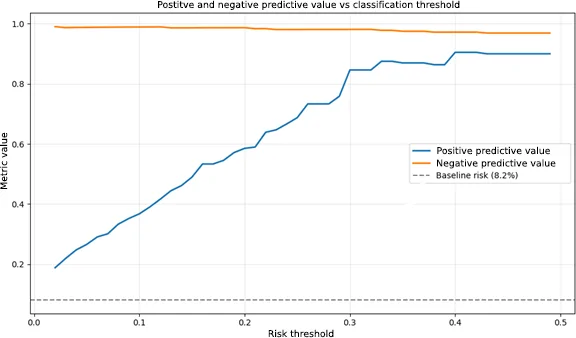
Analysis of PPV and NPV metrics depending on the classification threshold for model 9.

Analysis of the PPV and NPV curves showed that PPV increased as the classification threshold increased (Figure 8). At a classification threshold of 0.1, the PPV for IHM was approximately 37%, whereas at thresholds above 0.3, it exceeded 85%.

**Figure 9.**
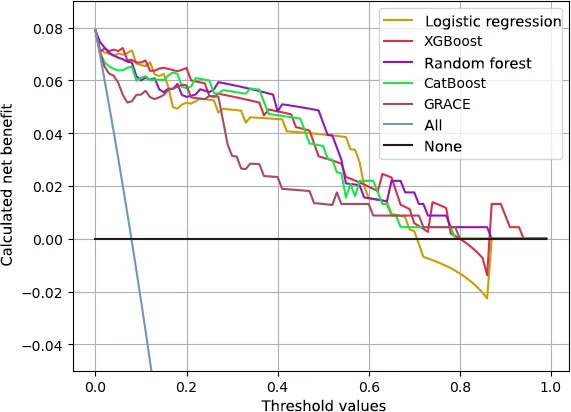
Decision curve analysis for evaluating the clinical utility of model 8. GRACE: Global Registry of Acute Coronary Events; XGBoost: Extreme Gradient Boosting.

Furthermore, DCA was used to compare the clinical utility of the models against the GRACE score (Figure 9). The analysis showed that all developed models provided greater clinical utility than the GRACE score, with the RF models demonstrating the highest net benefit.

**Figure 10.**
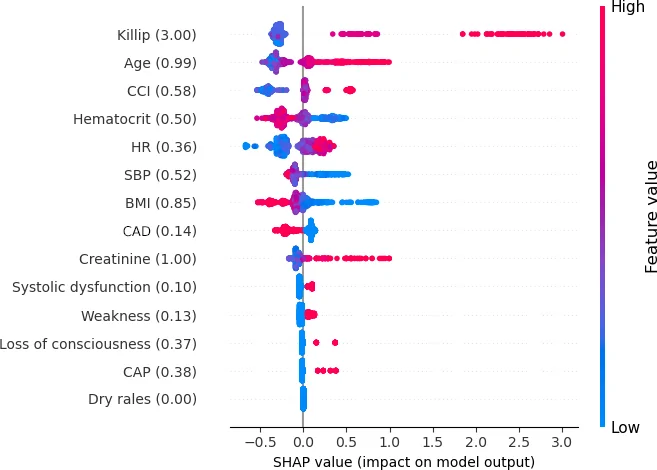
Contribution of predictors to the risk of in-hospital mortality for the Random Forest model during the diagnosis of non-ST–elevation acute coronary syndrome. CAD: coronary artery disease; CAP: community-acquired pneumonia; CCI: Charlson comorbidity index; HR: heart rate; SBP: systolic blood pressure; SHAP: Shapley Additive Explanations.

**Figure 11.**
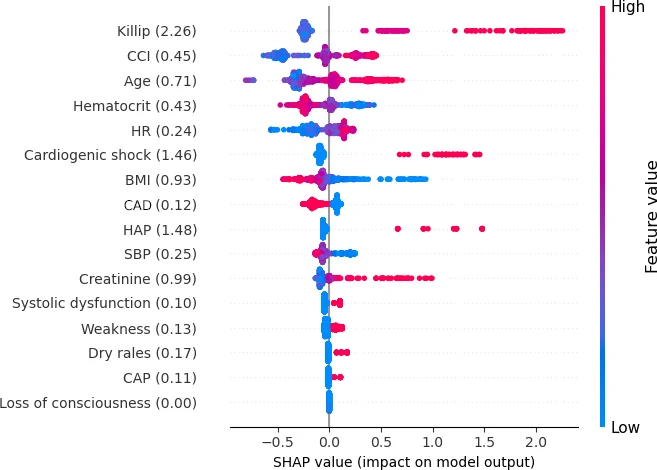
Contribution of predictors to the risk of in-hospital mortality for the Random Forest model during the diagnosis and treatment of non–ST-elevation acute coronary syndrome. CAD: coronary artery disease; CAP: community-acquired pneumonia; CCI: Charlson comorbidity index; HR: heart rate; SBP: systolic blood pressure; SHAP: Shapley Additive Explanations.

The importance of predictors used in the models was analyzed using the SHAP method. The results for the RF model are presented in Figure 10 (for model 8) and Figure 11 (for model 9).

Higher SHAP values, indicating an increased risk of IHM, were associated with pulmonary edema and cardiogenic shock at admission (Killip classes III and IV), older age, and greater comorbidity burden. Additional confirmed predictors included hematocrit and BMI, with lower values associated with a higher risk of IHM. During the treatment stage, predictors identified at earlier diagnostic stages remained important, while cardiogenic shock and HAP also emerged as significant contributors to mortality risk (Figure 11).

## Discussion

### Principal Findings

In this study, we proposed a stage-specific approach to predicting IHM in NSTE-ACS using ML algorithms that account for data availability at different phases of the diagnostic process. The developed models demonstrated high predictive performance, which consistently increased with the inclusion of additional clinical, laboratory, and instrumental predictors, achieving a maximum ROC-AUC of 0.951. RF-based models provided the best discrimination and clinical utility compared with traditional GRACE-based risk assessment.

Currently, NSTE-ACS remains the leading cause of emergency hospitalization in cardiology departments. Recently, due to the progressive aging of the population, the proportion of NSTE-ACS within the structure of acute coronary events has increased significantly. In 2020, the number of NSTE-ACS cases nearly doubled that of STEMI [25]. Despite improvements in the management of patients with NSTE-ACS, updated clinical guidelines, and high rates of invasive treatment application, real-world clinical practice acknowledges a lack of positive dynamics indicating further improvement in treatment outcomes for the main conditions within this syndrome [26]. For instance, according to data from the annual Swedish SWEDEHEART registry [1], a general trend of decreasing mortality from NSTEMI was observed between 1995 and 2008: IHM dropped from 12% to 4%, and long-term mortality from 27% to 16%. However, over the last 17 years (2008‐2024), the rates of in-hospital and 30-day mortality for NSTEMI have remained largely unchanged, reaching 3.6% and 4.5%, respectively, in 2023, despite an increased frequency of invasive treatment use [27]. In a large 3-year ACS registry presented by Toušek et al [28], IHM was 6.2% for NSTEMI and 1.2% for unstable angina.

Given the significant heterogeneity within the cohort of patients with NSTE-ACS, risk stratification for adverse cardiovascular events is essential, as this approach determines not only treatment strategy but also disease prognosis. It has been demonstrated that the use of risk stratification tools helps to avoid subjectivity in clinical decision-making for patients with NSTE-ACS [5,29].

Risk assessment and the subsequent determination of patient management strategy ensure the application of the necessary scope of established diagnostic and therapeutic methods in a hospital setting. According to a number of studies, this leads to improved short- and long-term clinical outcomes [30-32]. Furthermore, the quantitative calculation of the risk of adverse ischemic events surpasses subjective clinical assessment. Among existing calculators, the GRACE risk score remains the primary tool for objective risk stratification, both at admission and at discharge [33]. However, it is important to note several limitations of using this score with the cohort of contemporary patients with NSTE-ACS.

Primarily, 2 studies [13,14] that largely formed the basis for the original clinical guideline recommendations to implement and use the GRACE score in routine practice involved a patient population with a clinical profile significantly different from that of patients with contemporary NSTE-ACS. The modern profile is characterized by a significant comorbid status [6,34]. This feature logically reflects a global trend associated with the progressive aging of the population [25]. Furthermore, a recent large international multicenter study [16], which included a contemporary population of patients with NSTE-ACS, demonstrated that using GRACE score versions different from the original calculator leads to substantial variations in the classification of estimated risk, even when applying the same threshold values (ie, ≤108 and >140 points). This study also showed that the diagnostic performance of the 0/1-hour algorithm (using high-sensitivity cardiac troponin I) for identifying patients with myocardial infarction significantly surpasses that of any GRACE risk assessment used alone.

Thus, these limitations of the GRACE score, coupled with the persistent need to improve outcomes in NSTE-ACS—particularly long-term outcomes—necessitate the development of new risk calculators for adverse ischemic events, tailored to the modern clinical patient profile.

The results of our study not only confirmed several known facts but also revealed new insights. Within this research, multiple ML models were developed and compared for predicting IHM in a contemporary cohort of patients with NSTE-ACS. We confirmed the high prognostic value of the GRACE risk score (ROC-AUC=0.911, 95% CI 0.910‐0.913) and demonstrated that categorizing the continuous predictors of the GRACE score can improve predictive accuracy. Model 4, based on the continuous-form GRACE predictors, showed an ROC-AUC of 0.888 (95% CI 0.886‐0.890). The comparison of these models revealed a statistically significant difference (*P*<.001). Multilevel categorization of continuous predictors may enhance the predictive accuracy of prognostic models by incorporating additional knowledge that refines the relationships between predictors and the outcome [35]. Our experiments demonstrated that when a large number of additional predictors were included in the models, the difference between the model based on the GRACE score and the model incorporating the 5 individual predictors (age, HR, SBP, Killip class, and creatinine) became statistically nonsignificant.

Simultaneously, our study revealed that using additional anamnestic data and physical examination findings allows for the development of models with significantly superior predictive performance. This includes models for the first scenario, which do not incorporate laboratory results and can be applied at the very early stages of the diagnostic process (ROC-AUC=0.930, 95% CI 0.925‐0.935; sensitivity=0.843, 95% CI 0.828‐0.859; and specificity=0.852, 95% CI 0.847‐0.857). Expanding the predictor set with laboratory findings and subsequently with instrumental investigation results significantly enhanced the quality of IHM prediction in patients with NSTE-ACS, with the best model achieving an ROC-AUC of 0.942 (95% CI 0.937‐0.947), a sensitivity of 0.871 (95% CI 0.856‐0.887), and a specificity of 0.868 (95% CI 0.863‐0.873). The accuracy of IHM prediction during inpatient treatment reached an ROC-AUC of 0.951 (95% CI 0.946‐0.956), a sensitivity of 0.902 (95% CI 0.889‐0.916), and a specificity of 0.891 (95% CI 0.886‐0.896). High ROC-AUC, sensitivity, and specificity values indicate the strong discriminative performance of the developed models.

The best performance was demonstrated by models developed using RF, with ROC-AUC values ranging from 0.925 to 0.951 depending on the predictors used, indicating high predictive capability. The logistic regression model showed slightly lower performance, with ROC-AUC values ranging from 0.916 to 0.947 but remained a reliable predictive tool. The XGBoost and CB models demonstrated comparable performance, with ROC-AUC values ranging from 0.919 to 0.947. At the final diagnostic stage, model 7 demonstrated high predictive performance (ROC-AUC 0.942), although this was slightly lower than that of model 9, which incorporated treatment-period variables (ROC-AUC=0.951).

Given the substantial class imbalance in the analyzed dataset (IHM prevalence of 8.1%), additional evaluation metrics were applied, including the Brier score and PR-AUC, along with calibration analysis of the best-performing models and assessment of the relationship between PPV and NPV across classification thresholds. The analysis yielded the following findings. Model 1 demonstrated good discriminative performance (PR-AUC=0.62), low prediction error (Brier score=0.0378), minimal systematic bias (intercept −0.08), and a slight tendency to overestimate risk (slope 0.92). Model 6 showed improved discriminative ability (PR-AUC=0.641) and comparable prediction error (Brier score=0.0375) relative to model 1, with similarly minimal systematic bias (intercept=0.08) and a comparable tendency toward risk overestimation (slope 0.90). Model 9 demonstrated the best discriminative performance (PR-AUC=0.685) and the lowest prediction error (Brier score=0.0331), with no evidence of substantial miscalibration (slope 1.02), although a small intercept shift was observed (intercept=0.12). Overall, all models showed a favorable balance between discrimination and calibration and provided a wide range of predicted probabilities (approximately 80%), enabling effective risk stratification of patients at very high risk of IHM. On the basis of these findings, the developed models appear suitable for potential use in clinical practice.

To identify the model with the best predictive performance, DCA and SHAP analyses were additionally used. DCA showed that the RF model provided greater net benefit than all other models (Figure 9). Moreover, across all threshold probabilities, the net benefit of the RF model remained positive, indicating its potential clinical utility. SHAP analysis confirmed the importance of the GRACE score predictors for forecasting IHM in patients with NSTE-ACS (Figures 10 and 11). The highest feature importance was associated with the Killip class for AHF. At early stages, the most critical risk factors were elevated creatinine levels, advanced age, and high HR, while low SBP could be considered a marker for IHM. Concurrently, we demonstrated that low hematocrit levels, high CCI, and low BMI increase the risk of IHM. Interestingly, patients with a family history of CVD tolerated NSTE-ACS better than those without this factor. During the treatment phase, important risk factors were cardiogenic shock and HAP, while findings from the physical examination and SBP at admission, as well as LVSD and CAP, lost significance (Figure 11).

The SHAP analysis provides additional insight into the clinical mechanisms underlying the identified predictors of IHM. Factors such as cardiogenic shock and pulmonary edema (Killip classes III and IV) reflect severe acute hemodynamic compromise and are well-established markers of high short-term mortality risk in NSTE-ACS. Older age and a higher comorbidity burden likely reflect reduced physiological reserve and a higher prevalence of chronic organ dysfunction. Lower hematocrit and lower BMI may indicate anemia, frailty, or chronic disease states, which are associated with poorer tolerance of acute ischemic events.

From a clinical perspective, models developed for different stages of the diagnostic process may support early risk stratification and guide clinical decision-making. At the admission stage, early identification of high-risk patients may facilitate more intensive monitoring and earlier invasive evaluation. At later stages of hospitalization, identification of complications, such as cardiogenic shock or HAP, may guide escalation of supportive therapy and help prevent further clinical deterioration.

Recent studies have demonstrated the efficacy of ML algorithms, such as Support Vector Machine, XGBoost, and RF [36]. The ROC-AUC metric of the developed models ranged from 0.73 to 0.883. Similar accuracy results (ROC-AUC=0.87) for patients with NSTEMI were achieved using a multilayer perceptron, outperforming the GRACE score [37]. The RF method enabled the development of an IHM prognostic model for patients with NSTEMI with a ROC-AUC of 0.889 [17]. The RF method also provided the best IHM prediction results for patients with NSTEMI, with an ROC-AUC of 0.890 [19].

Our study demonstrated superior predictive accuracy, with ROC-AUCs of 0.942 during diagnosis and 0.951 during the treatment stage. Table 3 summarizes data reflecting the accuracy of various IHM prognostic models in patients with NSTE-ACS. For cross-study comparison, we limited the analysis to area under the curve, sensitivity, and specificity, as these metrics were the most consistently reported in previously published studies.

On the basis of the developed models, a web service—a calculator for assessing IHM risk in patients with NSTE-ACS—has been created and is available for clinical testing (the electronic link is provided in the supplementary materials in Multimedia Appendix 1).

**Table 3.** Comparative analysis of the accuracy of in-hospital mortality prognostic models in patients with non–ST-elevation acute coronary syndrome.

Researches	Predictors	ROC-AUC^a^	Sensitivity	Specificity
Model 7	Age, Killip class of acute heart failure, systolic blood pressure, heart rate, creatinine, hematocrit, BMI, community-acquired pneumonia, symptoms of weakness, Charlson comorbidity index, family history of cardiovascular diseases, dry rales, loss of consciousness, left ventricular systolic dysfunction	0.942	0.871	0.868
Model 9	Age, Killip class of acute heart failure, systolic blood pressure, heart rate, creatinine, hematocrit, BMI, community-acquired pneumonia, symptoms of weakness, Charlson comorbidity index, family history of cardiovascular diseases, dry rales, loss of consciousness, left ventricular systolic dysfunction, hospital-acquired pneumonia, cardiogenic shock	0.951	0.902	0.891
Cao and Li [19]	Length of hospital stay, acute physiology score III (SAPS 3), lactate dehydrogenase, Charlson comorbidity index, lactate, red cell distribution width, aspartate aminotransferase, prothrombin time, creatinine, count of atrial fibrillation episodes, angiotensin-converting enzyme inhibitors, chronic heart failure	0.890	0.8513	—^b^
Liu et al [38]	Discharge outcomes, mode of admission, communication skills, C-reactive protein, total cholesterol, high-density lipoprotein, low-density lipoprotein	0.749	0.625	0.781
Lee et al [17]	Age, cardiac arrest before hospitalization, hypertension, diastolic blood pressure, systolic blood pressure, diabetes mellitus, heart rate, cardiogenic shock, chronic heart failure, creatinine, hemoglobin	0.912	0.839	0.845
Kasim et al [36]	Age, sex, hypertension, history of myocardial infarction, chronic obstructive pulmonary disease, heart rate, systolic blood pressure, Killip class of acute heart failure, glucose, low-molecular-weight heparin, angiotensin II receptor blocker, beta-blocker	0.883	0.716	0.88
Kwon et al [37]	Killip class of acute heart failure, systolic blood pressure, glucose, heart rate, creatinine, sex, BMI, age, C-reactive protein, low-density lipoprotein, out-of-hospital cardiogenic shock, creatinine kinase-MB	0.87	—	—

a ROC-AUC: area under the receiver operating characteristic curve.

b Not applicable.

### Conclusions

The use of ML methods allowed the development of several prognostic models for IHM in patients with NSTE-ACS at various stages of the diagnostic process. Models based on the RF algorithm demonstrated the best predictive performance. We showed that incorporating additional anamnestic data and physical examination findings at hospital admission enables the development of a model surpassing the accuracy of the GRACE score (ROC-AUC=0.92 vs 0.911). The inclusion of laboratory data (creatinine and hematocrit) and instrumental findings (LVSD) further enhanced predictive accuracy (ROC-AUC=0.942). Risk factors recorded during inpatient treatment (HAP and cardiogenic shock) provided the highest prognostic accuracy, with an ROC-AUC of 0.951. The performance of the models and their potential clinical applicability were supported by calibration curve analysis as well as by the Brier score, PR-AUC, slope, and intercept metrics. The data obtained during this study were summarized in the form of model 9 and became the basis for the presented risk calculator. The application of this developed model in clinical practice could facilitate a personalized treatment approach and contribute to reducing IHM in patients with NSTE-ACS.

### Limitations

To confirm the efficacy of the developed models, further research is required, including prospective and multicenter trials. Future work should also explore the integration of these models into clinical decision support systems for automated data analysis and improved quality of care.

## Supplementary material

10.2196/89242 Multimedia Appendix 1 Supplementary tables presenting baseline clinical characteristics, variables excluded because of missing data, and the predictive performance (coefficients and ROC-AUC values) of individual variables included in the machine learning analysis.

## Abbreviations

AHF: acute heart failure
CAD: coronary artery disease
CAP: community-acquired pneumonia
CB: CatBoost
CCI: Charlson comorbidity index
CHF: chronic heart failure
CKD: chronic kidney disease
CRUSADE: Can Rapid Risk Stratification of Unstable Angina Patients Suppress Adverse Outcomes With Early Implementangina Patients Suppress Adverse Outcomes With Early Implementation
CVD: cardiovascular diseases
DBP: diastolic blood pressure
DCA: decision curve analysis
ECG: electrocardiography
EchoCG: echocardiography
GRACE: Global Registry of Acute Coronary Events
HAP: hospital-acquired pneumonia
HR: heart rate
ICA: invasive coronary angiography
IHM: in-hospital mortality
LVEF: left ventricular ejection fraction
LVSD: left ventricular systolic dysfunction
ML: machine learning
MLR: multivariate logistic regression
NPV: negative predictive value
NSTE-ACS: non–ST-elevation acute coronary syndrome
NSTEMI: non–ST-segment elevation myocardial infarction
OR: odds ratio
PPV: positive predictive value
PR-AUC: area under the precision-recall curve
QTc: corrected interval QT
RF: Random Forest
ROC-AUC: area under the receiver operating characteristic curve
SBP: systolic blood pressure
SHAP: Shapley Additive Explanations
STEMI: ST-segment elevation myocardial infarction
SV: stroke volume
ULR: univariate logistic regression
XGBoost: Extreme Gradient Boosting
