# Supplementary material for: Machine Learning–Based Risk Prediction of In-Hospital Mortality in Patients With Non–ST-Elevation Acute Coronary Syndrome at Various Stages of the Diagnostic Process: Observational Study
Source: JMIR Med Inform. 2026 Aug 19;14:e89242. doi: 10.2196/89242 (PMC13489111; doi:10.2196/89242)
Supplement: Supplementary file 1 [file medinform-v14-e89242-s001.docx]

**Multimedia Appendix 1.** Supplementary tables presenting baseline clinical characteristics, variables excluded because of missing data, and the predictive performance (coefficients and ROC-AUC values) of individual variables included in the machine learning analysis.

**Table S1 -** Results of intergroup comparisons of main clinical and functional parameters

| **Parameters** | **All patients, n=1144** | **Surviving patients, n=1050** | **Deceased patients, n=94** | **OR; [95%] CI** | ***P*-value** | **Treatment stage^a^** |
| --- | --- | --- | --- | --- | --- | --- |
| **Demographic and anthropometric indicators** | | | | | | |
| Age, years | 67 (59.75; 75) | 66 (59; 74) | 80.5 (72; 85) | - | <.001 | 1 |
| Sex: Female, n (%) | 487 (42.6%) | 438 (41.7%) | 49 (52.1%) | 1.521 [0.997; 2.322] | 0.065 | 1 |
| Sex: Male, n (%) | 657 (57.4%) | 612 (58.3%) | 45 (47.9%) |  |  | 1 |
| Body mass index, kg/m^2^ | 28.31 (25.18; 32.3) | 28.59 (25.5; 32.4) | 25.95 (22.925; 29.238) | - | <.001 | 1 |
| **Patients' condition on admission** | | | | | | |
| SBP, mmHg | 138 (120; 154) | 139.5 (123; 155) | 110.5 (99.25; 135.75) | - | <.001 | 1 |
| DBP, mmHg | 80 (70; 90) | 80 (70; 90) | 69 (59.25; 80) | - | <.001 | 1 |
| HR, bpm | 76 (65; 90) | 75 (65; 88) | 90 (75.25; 108.75) | - | <.001 | 1 |
| Killip class of acute heart failure | 1 (1; 1) | 1 (1; 1) | 4 (1; 4) | - | 0 | 1 |
| Acute heart failure (Killip class II-IV),  n (%) | 175 (15.3%) | 112 (10.7%) | 63 (67%) | 17 [10; 6; 27.3] | 0 | 1 |
| Acute heart failure (Killip class III-IV), n (%) | 130 (11.4%) | 67 (6.4%) | 63 (67%) | 29.8 [18.2; 49] | 0 | 1 |
| Community-acquired pneumonia, n (%) | 42 (3.7%) | 25 (2.38%) | 17 (18.09%) | 9.05 [4.69; 17.48] | <.001 | 1 |
| Cardiac arrhythmias, n (%) | 463 (40.47%) | 419 (39.9%) | 44 (46.8%) | 1.33 [0.87; 2.02] | 0.231 | 1 |
| Anginal pain, n (%) | 366 (32%) | 334 (31.8%) | 32 (34%) | 1.1 [0.71; 1.73] | 0.742 | 1 |
| Dyspnea, n (%) | 239 (21%) | 192 (18.29%) | 47 (50.0%) | 4.47 [2.9; 6.89] | <.001 | 1 |
| Symptoms of weakness, n (%) | 209 (18.3%) | 172 (16.38%) | 37 (39.36%) | 3.31 [2.12; 5.17] | <.001 | 1 |
| Dizziness, n (%) | 73 (6.4%) | 58 (5.52%) | 15 (15.96%) | 3.25 [1.76; 5.99] | <.001 | 1 |
| Loss of consciousness, n (%) | 34 (2.97%) | 20 (1.9%) | 14 (14.9%) | 9.02 [4.39; 18.51] | <.001 | 1 |
| Severe or critical condition, n (%) | 127 (11.1%) | 80 (7.6%) | 47 (50%) | 12.125 [7.62; 19.29] | 0 | 1 |
| Pulmonary rales, n (%) | 146 (12.8%) | 110 (10.5%) | 36 (38.3%) | 5.3 [3.35; 8.41] | <.001 | 1 |
| Dry rales, n (%) | 53 (4.6%) | 41 (4%) | 11 (11.7%) | 3.18 [1.58; 6.4] | 0.002 | 1 |
| Peripheral edema, n (%) | 98 (8.6%) | 91 (8.7%) | 7 (7.4%) | 0.85 [0.38; 1.89] | 0.832 | 1 |
| GRACE, scores | 109 (90; 131) | 108 (88; 125) | 188.5 (147; 211.75) | - | 0 | 2 |
| Cardiogenic shock, n (%) | 54 (4.7%) | 13 (1.2%) | 41 (43.6%) | 61.7 [31.2; 122.1] | 0 | 1 |
| **Anamnestic data** | | | | | | |
| Hypotensive episodes, n (%) | 28 (2.4%) | 11 (1%) | 17 (18.1%) | 20.85 [9.44; 46.08] | 0 | 1 |
| Chronic kidney disease, n (%) | 448 (39.2%) | 386 (36.8%) | 62 (66%) | 3.33 [2.13; 5.2] | <.001 | 1 |
| Chronic kidney disease, stages II–IV, n (%) | 228 (19.9%) | 180 (17.1%) | 48 (51.1%) | 5.04 [3.26; 7.79] | <.001 | 1 |
| Chronic kidney disease, stages III–IV, n (%) | 93 (8.1%) | 62 (5.9%) | 31 (33%) | 7.8 [4.75; 12.93] | <.001 | 1 |
| Chronic kidney disease, stage IV, n (%) | 21 (1.8%) | 9 (0.86%) | 12 (12.8%) | 16.9 [6.9; 41.3] | <.001 | 1 |
| Malignant neoplasm, n (%) | 68 (5.9%) | 57 (5.4%) | 11 (11.7%) | 2.3 [1.17; 4.57] | 0.025 | 1 |
| Peripheral arterial disease, n (%) | 68 (5.9%) | 62 (5.9%) | 6 (6.38%) | 1.09 [0.46; 2.58] | 1 | 1 |
| Chronic heart failure, NYHA functional class III-IV, n (%) | 117 (10.2%) | 90 (8.57%) | 27 (28.7%) | 4.3 [2.62; 7.06] | <.001 | 1 |
| Myocardial infarction, n (%) | 455 (39.8%) | 402 (38.3%) | 41 (43.6%) | 2.08 [1.36; 3.19] | <.001 | 1 |
| Valvular heart disease, n (%) | 105 (9.18%) | 93 (8.86%) | 12 (12.77%) | 1.51 [0.79; 2.86] | 0.284 | 1 |
| Atrial fibrillation, n (%) | 245 (21.41%) | 216 (20.57%) | 29 (30.85%) | 1.72 [1.08; 2.74] | 0.028 | 1 |
| PCI, n (%) | 282 (24.65%) | 265 (25.24%) | 15 (18.08%) | 0.654 [0.38; 1.13] | 0.157 | 1 |
| CABG, n (%) | 92 (8.04%) | 88 (8.38%) | 4 (4.26%) | 0.49 [0.17; 1.35] | 0.226 | 1 |
| Stroke, n (%) | 102 (8.9%) | 90 (8.57%) | 12 (12.77%) | 1.56 [0.82; 2.97] | 0.239 | 1 |
| Diabetes mellitus type 2, n (%) | 270 (23.6%) | 240 (22.86%) | 30 (31.9%) | 1.58 [1; 2.5] | 0.064 | 1 |
| Current or former smoking, n (%) | 499 (43.6%) | 475 (45.2%) | 24 (25.5%) | 0.42 [0.257; 0.67] | <.001 | 1 |
| Family history of cardiovascular diseases, n (%) | 455 (39.78%) | 439 (41.8%) | 16 (17%) | 0.285 [0.164; 0.496] | <.001 | 1 |
| Charlson comorbidity index, scores | 6 (4; 8) | 5.0 (3.0; 7.0) | 8.0 (6.0; 10.0) | - | <.001 | 1 |
| **Laboratory tests on admission** | | | | | | |
| Leukocytes, 10^9/L | 8.4 (6.75; 10.49) | 8.29 (6.7; 10.05) | 10.7 (7.68; 14) | - | <.001 | 2 |
| Hemoglobin, g/L | 135 (121; 148) | 136 (123; 148) | 113 (99; 132) | - | <.001 | 2 |
| Erythrocytes, 10^12/L | 4.6 (4.19; 4.99) | 4.6 (4.25; 5) | 4.2 (3.57; 4.6) | - | <.001 | 2 |
| Hematocrit, % | 39 (35; 42.5) | 39,85 (36,0; 43,0) | 34,0 (30,0; 39,0) | - | <.001 | 2 |
| Platelets, 10^9/L | 224.5 (183;265.75) | 224 (184; 264) | 228 (170; 296) |  | 0.689 | 2 |
| Erythrocyte sedimentation rate, mm/hr | 12 (5.5; 21) | 17 (9; 34) | 12 (5; 21) | - | 0.004 | 2 |
| Total protein, g/L | 70 (65.6; 74) | 70.1 (66; 74) | 65 (57.175; 71) | - | <.001 | 2 |
| Glucose, mmol/L | 7 (6; 8.7) | 6.9 (6; 8.5) | 8.75 (6.9; 11.125) | - | <.001 | 2 |
| Creatinine, µmol/L | 95 (82; 119) | 94 (81; 115) | 129 (101; 189) | - | <.001 | 2 |
| Glomerular filtration rate (CKD-EPI), mL/min/1.73 m² | 66 (47; 82) | 68 (50.63; 84) | 39 (23; 55) | - | 0 | 2 |
| Total bilirubin, µmol/L | 12.6 (9.2; 17.7) | 12.6 (9.125; 17.475) | 13.65 (9.425; 19.675) | - | 0.276 | 2 |
| AST, U/L | 25.7 (19; 39.7) | 25 (19; 38) | 39 (23.425; 100) | - | <.001 | 2 |
| ALT, U/L | 22 (15.7; 34) | 22 (16; 33) | 21.15 (13.175; 58) | - | 0.385 | 2 |
| Creatine kinase, U/L | 120 (78; 213) | 119 (77; 200) | 202 (95.25; 499.5) | - | <.001 | 2 |
| C-reactive protein, mg/L | 4.55 (2.6; 22.15) | 4.2 (2.45; 19.1) | 22 (8.25; 95.9) | - | <.001 | 2 |
| Troponin-I elevation, n (%) | 330 (28.8%) | 268 (25.5%) | 62 (66%) | 7.24 [4.24; 12.36] | <.001 | 2 |
| Total cholesterol, mmol/L | 4.55 (3.7; 5.56) | 4.6 (3.75; 5.6) | 4 (3; 5.425) | - | <.001 | 2 |
| Triglycerides, mmol/L | 1.2 (0.8; 1.74) | 1.2 (0.8; 1.76) | 1.14 (0.89; 1.62) | - | 0.990 | 2 |
| LDL-C, mmol/L | 2.715 (1.9; 3.6) | 2.8 (1.9; 3.565) | 2.3 (1.6; 3.7) | - | 0.063 | 2 |
| HDL-C, mmol/L | 1.21 (0.993; 1.5) | 1.22 (1; 1.5) | 1.11 (0.88; 1.4) | - | 0.014 | 2 |
| Fibrinogen, g/L | 3.3 (3; 4) | 3.3 (3; 3.9) | 3.49 (2.9; 4.3) | - | 0.218 | 2 |
| International Normalized Ratio, ratio | 1 (0.96; 1.1) | 1 (0.96; 1.1) | 1.1 (1; 1.275) | - | <.001 | 2 |
| **Instrumental examinations** | | | | | | |
| SpO2 (%) | 97 (95; 98) | 94 (88; 96) | 97 (96; 98) | - | <.001 | 1 |
| Left ventricular ejection fraction (B-mode), % | 59 (50; 64) | 60 (52; 64) | 44 (35; 59) | - | <.001 | 3 |
| Left atrium, mm | 40 (37; 44) | 40 (37; 44) | 42 (38; 46) | - | 0.121 | 3 |
| Right ventricle, mm | 25 (23; 26) | 25 (23; 36) | 25 (23; 26) | - | 0.402 | 3 |
| Ascending aorta, mm | 34 (32; 37) | 34 (32; 37) | 36 (33; 39) | - | 0.051 | 3 |
| Interventricular septum, mm | 11 (10; 12) | 11 (10; 12) | 11 (10; 13) | - | 0.544 | 3 |
| Left ventricular posterior wall, mm | 10.8 (10; 11.8) | 10.8 (10; 11.5) | 11 (10; 12) | - | 0.218 | 3 |
| Myocardial mass, g | 201 (168; 240) | 201 (168; 239) | 209 (164; 255) | - | 0.420 | 3 |
| Left ventricular mass index, g/m² | 104 (90; 124) | 104 (90; 122.5) | 115 (103; 147) | - | 0.002 | 3 |
| Left ventricular end-diastolic diameter, mm | 48 (45; 52) | 48 (45; 52) | 47 (42.5; 53) | - | 0.510 | 3 |
| Left ventricular end-systolic diameter, mm | 32 (30; 37) | 32 (30; 37) | 36 (31; 42) | - | 0.232 | 3 |
| Left ventricular end-diastolic volume, ml | 105 (87; 126) | 105 (81.2; 126) | 97.5 (78.25; 134.5) | - | 0.257 | 3 |
| Left ventricular end- systolic volume, ml | 42 (33; 58.25) | 42 (33; 57) | 51 (35.5; 89.5) | - | 0.014 | 3 |
| Stroke volume, ml | 60 (50; 69) | 60.0 (51.0; 69.0) | 45.5 (37.75; 55.25) | - | <.001 | 3 |
| Right ventricular systolic pressure, mmHg | 30 (26; 38) | 30 (26; 36) | 47 (36; 55) | - | <.001 | 3 |
| E/A ratio | 0.77 (0.63; 1.19) | 0.76 (0.63; 1.165) | 1.04 (0.6; 1.49) | - | 0.111 | 3 |
| Left ventricular systolic dysfunction, n (%) | 215 (18.8%) | 171 (16.3%) | 44 (46.8%) | 4.6 [3; 7.16] | <.001 | 3 |
| Left ventricular diastolic dysfunction, n (%) | 778 (68%) | 714 (68%) | 64 (68.1%) | 1.5 [0.9; 2.53] | 0.150 | 3 |
| Left ventricular hypertrophy, n (%) | 416 (36.4%) | 378 (36%) | 38 (40.4%) | 1.35 [0.87; 2.1] | 0.219 | 3 |
| Regional contractility disorders, n (%) | 578 (50.5%) | 515 (49%) | 63 (67%) | 2.19 [1.39; 3.43] | <.001 | 3 |
| Interval QRS, ms | 98 (88; 112) | 98 (88; 110) | 108 (94; 130) | - | <.001 | 1 |
| Interval QT, ms | 390 (362; 418) | 390 (362; 418) | 384 (352; 414) | - | 0.326 | 1 |
| Corrected interval QT, ms | 420 (402; 441) | 419 (402; 439) | 433 (413; 461) | - | <.001 | 1 |
| Second- and third-degree atrioventricular block, n (%) | 331 (28.9%) | 298 (28.4%) | 33 (35.1%) | 1.37 [0.88; 2.13] | 0.208 | 1 |
| Multivessel coronary artery disease, n (%) | 346 (30.2%) | 304 (28.9%) | 37 (44.7%) | - | <.001 | 4 |
| Angiographic evidence of slow coronary flow, n (%) | 203 (17.7%) | 200 (19%) | 3 (3.2%) | 0.14 [0.044; 0.447] | <.001 | 4 |
| **In-hospital complications** | | | | | | |
| Hospital-acquired pneumonia, n (%) | 35 (3.06%) | 19 (1.8%) | 16 (17%) | 11.1 [5.5; 22.5] | <.001 | 4 |
| Signs of ongoing myocardial ischemia on admission, n (%) | 160 (14%) | 109 (10.4%) | 51 (54.3%) | 10.24 [6.52; 16.08] | 0 | 1 |
| Sepsis, n (%) | 9 (0.79%) | 2 (0.19%) | 7 (7.4%) | 42.2 [8.6; 206] | <.001 | 4 |
| Stroke, n (%) | 15 (1.3%) | 7 (0.67%) | 8 (8.5%) | 13.86 [4.9; 39.1] | <.001 | 4 |
| ALT – alanine aminotransferase; AST – aspartate aminotransferase; CABG – coronary artery bypass grafting; CI – confidence intervals; DBP – diastolic blood pressure; HDL-C – high-density lipoprotein cholesterol; HR – heart rate; LDL-C – low-density lipoprotein cholesterol; OR – odds ratios; PCI – percutaneous coronary intervention; SBP – systolic blood pressure.  **Treatment stage^a^:** 1 – admission; 2 – after laboratory tests; 3 – after receiving the echocardiography data; 4 – during the hospitalization. | | | | | | |

**Table S2 -** Variables excluded from the study due to more than 10% missing data.

| **Predictors** | **Not null (%)** | **Treatment stage** |
| --- | --- | --- |
| **Coronary CT angiography (prior to hospitalization)** | **539 (47%)** | **Stage 1.** Admission |
| **Stress test (prior to hospitalization)** | **539 (47%)** | **Stage 1.** Admission |
| **Hemoglobin, g/L (repeat measurement during hospitalization)** | **810 (71%)** | **Stage 4.** During the hospitalization |
| **Creatinine, µmol/L (repeat measurement during hospitalization)** | **866 (75.7%)** | **Stage 4.** During the hospitalization |
| **Serum iron, µmol/L** | **153 (13.4%)** | **Stage 2.** After laboratory tests |
| **Glycated hemoglobin (HbA1c), %** | **216 (18.9%)** | **Stage 2.** After laboratory tests |
| **Six-minute walk test, meters** | **720 (63%)** | **Stage 4.** During the hospitalization |

**Table S3** - Weight coefficients and ROC AUC for isolated predictors

| **Sign** | **Coefficient** | **AUC** | ***P*-value** |
| --- | --- | --- | --- |
| GRACE | 7.107 [7.093, 7.121] | 0.907 [0.901, 0.913] | 0 |
| Killip class of acute heart failure | 4.576 [4.562, 4.589] | 0.805 [0.794, 0.815] | 0 |
| Glomerular filtration rate | -4.945 [-4.959, -4.930] | 0.799 [0.789, 0.809] | 0 |
| Acute heart failure (Killip class III-IV) | 3.190 [3.181, 3.200] | 0.798 [0.788, 0.808] | 0 |
| Age | 4.52 [4.5; 4.54] | 0.793 [0.784, 0.802] | <.001 |
| Right ventricular systolic pressure | 3.093 [3.076, 3.110] | 0.786 [0.775, 0.798] | <.001 |
| Acute heart failure (Killip class II-IV) | 2.677 [2.668, 2.686] | 0.776 [0.767, 0.786] | 0 |
| SpO_2_ | -4.026 [-4.040, -4.011] | 0.774 [0.764, 0.783] | 0 |
| Stroke volume | -3.606 [-3.625, -3.587] | 0.774 [0.762, 0.786] | <.001 |
| Charlson comorbidity index | 3.840 [3.824, 3.856] | 0.769 [0.760, 0.778] | <.001 |
| C-reactive protein | 2.253 [2.232, 2.273] | 0.755 [0.742, 0.769] | <.001 |
| Systolic blood pressure | -4.207 [-4.228, -4.186] | 0.747 [0.734, 0.759] | <.001 |
| Creatinine | 3.922 [3.897, 3.947] | 0.747 [0.736, 0.759] | <.001 |
| Hemoglobin | -3.361 [-3.378, -3.344] | 0.735 [0.724, 0.746] | <.001 |
| Troponin-I elevation | 1.841 [1.832, 1.850] | 0.722 [0.713, 0.731] | <.001 |
| Left ventricular ejection fraction (B-mode) | -3.458 [-3.474, -3.442] | 0.721 [0.709, 0.734] | <.001 |
| Hematocrit | -3.418 [-3.439, -3.397] | 0.718 [0.706, 0.730] | <.001 |
| Signs of ongoing myocardial ischemia within the first 24 hours | 2.209 [2.200, 2.218] | 0.712 [0.701, 0.723] | 0 |
| Multivessel coronary artery disease | 1.547 [1.537, 1.558] | 0.707 [0.694, 0.721] | <.001 |
| Severe or critical condition | 2.358 [2.348, 2.368] | 0.706 [0.696, 0.716] | 0 |
| Diastolic blood pressure | -3.801 [-3.822, -3.780] | 0.705 [0.693, 0.718] | <.001 |
| Chronic kidney disease | 2.594 [2.580, 2.608] | 0.699 [0.687, 0.710] | <.001 |
| Leukocytes | 3.619 [3.597, 3.641] | 0.693 [0.679, 0.707] | <.001 |
| Total protein | -3.286 [-3.312, -3.260] | 0.689 [0.671, 0.707] | <.001 |
| Glucose | 2.054 [2.035, 2.073] | 0.683 [0.671, 0.695] | <.001 |
| Heart rate | 2.905 [2.884, 2.926] | 0.680 [0.667, 0.693] | <.001 |
| Chronic kidney disease, stages II-IV | 1.541 [1.532, 1.550] | 0.667 [0.658, 0.676] | <.001 |
| Erythrocytes | -3.008 [-3.030, -2.986] | 0.666 [0.653, 0.679] | <.001 |
| Body mass index | -2.504 [-2.526, -2.483] | 0.665 [0.653, 0.677] | <.001 |
| International Normalized Ratio | 1.568 [1.549, 1.588] | 0.664 [0.652, 0.676] | <.001 |
| Aspartate aminotransferase | 2.361 [2.342, 2.380] | 0.656 [0.641, 0.671] | <.001 |
| Dyspnea | 1.428 [1.419, 1.438] | 0.654 [0.643, 0.664] | <.001 |
| Chronic kidney disease, stages III-IV | 1.929 [1.918, 1.940] | 0.637 [0.628, 0.647] | <.001 |
| Interval QRS | 1.673 [1.655, 1.691] | 0.635 [0.622, 0.648] | <.001 |
| Creatine kinase | 1.605 [1.585, 1.625] | 0.631 [0.616, 0.646] | <.001 |
| Pulmonary rales | 1.589 [1.579, 1.599] | 0.630 [0.620, 0.640] | <.001 |
| Family history of cardiovascular diseases | -1.157 [-1.169, -1.146] | 0.621 [0.614, 0.629] | <.001 |
| Left ventricular mass index | 1.622 [1.605, 1.640] | 0.621 [0.607, 0.636] | <.001 |
| Total cholesterol | -1.495 [-1.520, -1.471] | 0.614 [0.600, 0.628] | 0.002 |
| Symptoms of weakness | 1.135 [1.125, 1.145] | 0.614 [0.602, 0.625] | <.001 |
| Erythrocyte sedimentation rate | 1.658 [1.636, 1.679] | 0.607 [0.587, 0.628] | <.001 |
| Left ventricular end-systolic volume | 1.578 [1.559, 1.596] | 0.605 [0.587, 0.624] | <.001 |
| Corrected interval QT | 1.486 [1.464, 1.509] | 0.605 [0.592, 0.618] | 0.001 |
| Chronic heart failure, NYHA functional class III-IV | 1.370 [1.362, 1.379] | 0.597 [0.588, 0.606] | <.001 |
| High-density lipoprotein cholesterol | -1.426 [-1.448, -1.404] | 0.594 [0.579, 0.609] | 0.005 |
| Current or former smoking | -0.828 [-0.837, -0.820] | 0.592 [0.582, 0.602] | 0.001 |
| Regional contractility disorders | 0.740 [0.731, 0.750] | 0.592 [0.582, 0.602] | 0.001 |
| Hypotensive episodes | 2.598 [2.585, 2.610] | 0.584 [0.575, 0.592] | <.001 |
| Myocardial infarction | 0.702 [0.694, 0.709] | 0.582 [0.572, 0.591] | 0.001 |
| Angiographic evidence of slow coronary flow | -1.518 [-1.532, -1.504] | 0.580 [0.576, 0.584] | 0.001 |
| Community-acquired pneumonia | 1.972 [1.959, 1.984] | 0.573 [0.566, 0.581] | <.001 |
| Chronic kidney disease, stages IV | 2.323 [2.306, 2.340] | 0.561 [0.554, 0.567] | <.001 |
| Loss of consciousness | 1.923 [1.908, 1.938] | 0.566 [0.559, 0.574] | <.001 |
| Atrial fibrillation | 0.509 [0.499, 0.519] | 0.556 [0.547, 0.566] | 0.021 |
| Dizziness | 1.064 [1.051, 1.077] | 0.549 [0.541, 0.557] | <.001 |
| Dry rales | 1.015 [1.001, 1.029] | 0.538 [0.532, 0.544] | 0.001 |
| Malignant neoplasm | 0.724 [0.708, 0.741] | 0.534 [0.527, 0.541] | 0.016 |
